# Workflow for shake flask and plate cultivations with fats for polyhydroxyalkanoate bioproduction

**DOI:** 10.1007/s00253-023-12599-w

**Published:** 2023-06-02

**Authors:** Sebastian L. Riedel, Ewelina N. Donicz, Paula Ferré-Aparicio, Lara Santolin, Anna-Maria Marbà-Ardébol, Peter Neubauer, Stefan Junne

**Affiliations:** 1 Institute of Biotechnology, Chair of Bioprocess Engineering, Technische Universität Berlin, Ackerstraße 76 ACK 24, D-13355 Berlin, Germany; 2 Department VIII – Mechanical Engineering, Event Technology and Process Engineering, Laboratory of Environmental and Bioprocess Engineering, Berliner Hochschule für Technik, Seestr. 64, Berlin, D-13347 Germany; 3 Department of Chemistry and Bioscience, Aalborg University Esbjerg, Niels Bohrs Vej 8, DK-6700 Esbjerg, Denmark

**Keywords:** Shake flask design, Microwell plates, Parallel cultivation, Bioprocess development, Waste animal fats, Polyhydroxyalkanoate

## Abstract

**Abstract:**

Since natural resources for the bioproduction of commodity chemicals are scarce, waste animal fats (WAF) are an interesting alternative biogenic residual feedstock. They appear as by-product from meat production, but several challenges are related to their application: first, the high melting points (up to 60 °C); and second, the insolubility in the polar water phase of cultivations. This leads to film and clump formation in shake flasks and microwell plates, which inhibits microbial consumption. In this study, different flask and well designs were investigated to identify the most suitable experimental set-up and further to create an appropriate workflow to achieve the required reproducibility of growth and product synthesis. The dissolved oxygen concentration was measured in-line throughout experiments. It became obvious that the gas mass transfer differed strongly among the shake flask design variants in cultivations with the polyhydroxyalkanoate (PHA) accumulating organism *Ralstonia eutropha*. A high reproducibility was achieved for certain flask or well plate design variants together with tailored cultivation conditions. Best results were achieved with bottom baffled glass and bottom baffled single-use shake flasks with flat membranes, namely, >6 g L^-1^ of cell dry weight (CDW) with >80 wt% polyhydroxybutyrate (PHB) from 1 wt% WAF. Improved pre-emulsification conditions for round microwell plates resulted in a production of 14 g L^-1^ CDW with a PHA content of 70 wt% PHB from 3 wt% WAF. The proposed workflow allows the rapid examination of fat material as feedstock, in the microwell plate and shake flask scale, also beyond PHA production.

**Key points:**

• *Evaluation of shake flask designs for cultivating with hydrophobic raw materials*

• *Development of a workflow for microwell plate cultivations with hydrophobic raw materials*

• *Production of polyhydroxyalkanoate in small scale experiments from waste animal fat*

**Graphical Abstract:**

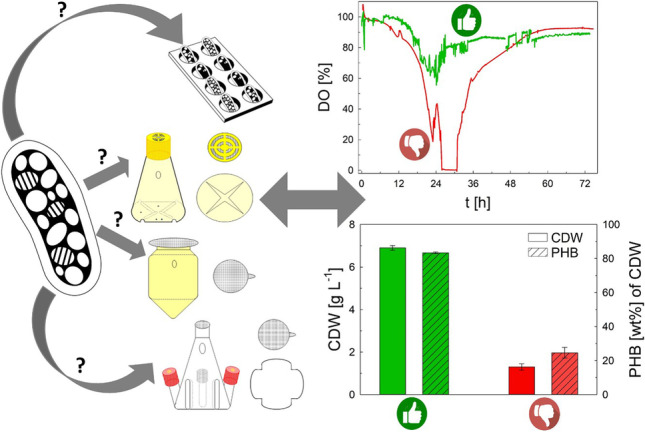

**Supplementary Information:**

The online version contains supplementary material available at 10.1007/s00253-023-12599-w.

## Introduction

Polyhydroxyalkanoates (PHAs) are intracellularly stored polyesters which are synthesized from many microorganisms. They are biodegradable and biocompatible substitutes to petroleum-based plastics (Wang and Chen 2017; Meereboer et al. 2020). Beside recovery, the costs of the carbon feedstock is the most important cost factor for industrial production (Riedel and Brigham 2019; da Cruz Pradella 2020). According to Toldrá-Reig et al., about one third of the waste animal fats (WAF) are residuals from Europe’s meat industry. They are used as substrate in biodiesel and biogas production (Toldrá-Reig et al. 2020). Low quality WAF is a promising feedstock for PHA production. They are inexpensive with respect to their carbon content compared to sugar-based substrates (Riedel and Brigham 2020; Gutschmann et al. 2022). WAF from tallow and from bones are suitable to be used as bioliquids after a tailor-made treatment for free fatty acid removal (Rosson et al. 2021). It is also technically feasible to convert them into triglycerides and add them to biodiesel (Ndiaye et al. 2020). While commercial application lacks economic competitiveness (Mata et al. 2018), research is conducted to identify new enzymes and process design until recently (Martins et al. 2021). This might open new opportunities for commercialization (Mata et al. 2018) through the use of microbial processes (Cheng et al. 2021). Enzymatic esterification of the free fatty acids with lipases represents a potential pre-treatment method (Martins et al. 2021). A comparison with an alternative use in incineration proved that rendering opens economic perspectives (Zagklis et al. 2020). Low-quality WAF application without the requirement of pretreating the free fatty acid fraction for *Ralstonia eutropha* cultivations was demonstrated (Saad et al. 2021; Gutschmann et al. 2023a). The key enzyme to ensure emulsification and subsequent consumption of fatty acids is a secreted lipase (Lu et al. 2013). Nevertheless, working with WAF remains challenging, as they have mostly high melting temperatures up to 60 °C. They form a heterogeneous mixture in aqueous media. WAF, which are encountered in film formation at the walls of cultivation systems, are not accessible to *R. eutropha*. This might lead to poor reproducibility and growth and certainly to erroneous yield quantification. In the case of *R. eutropha*, issues in bioreactor cultivations were overcome by using non-convertible emulsifiers such as gum arabic (Riedel et al. 2015) or by developing tailored feeding and pre-culture conditions without the need of emulsifiers (Gutschmann et al. 2023a; Gutschmann et al. 2023b). Due to the cost pressure for PHA production and the ability of *R. eutropha* to degrade fats, WAF are attractive substrates for an economical PHA production.

Parallel small-scale cultivation systems like shake flasks and microwell plates became common tools for an accelerated and automated bioprocess development in the laboratory. One drawback, however, is the huge sensitivity of these systems to insoluble components like fats. They are usually not well mixed with the core of the liquid phase due to natural phase separation in a laminar fluid flow or film formation at the typically unipolar plastic vessel walls. Additionally, lipophilic substances might clock the pores of the filter covers’ membranes. This effect drastically reduces the gas mass transfer with the environment. The importance of lipophilic components that are used as substrates to produce fine and bulk biochemical compounds is, however, rising in parallel to intensified efforts for a bio-based economy. Therefore, investigations and optimizations are required to improve the cultivation performance with these substrates in shake flask and microwell plate cultivations. As far as we know, no protocols are available yet for the cultivation of microorganisms with WAF in the microwell plate scale. The influence of different shake flask designs has not been investigated either.

The determination of optimal operation settings is, however, complex due to the high degree of interaction between different factors. A laminar flow and spout formation will contribute to a comparably high k_L_a-value (Maier et al. 2004) or a turbulent flow may lead to a faster formation of a well-mixed emulsion. In both cases, a lipid layer may form at the vessel walls, and it is not easily accessible as nutrient for the microbes. Due to the lack of data, standard shake flasks are often the first choice, regardless of the specific needs for certain applications. This can be requirements about the oxygen demand or the complex multiphasic media components. The options various single-use and reusable equipment offer in the lab scale are hardly explored, although the choice of a suitable design can certainly improve the cultivation performance as shown for several microbial cultivations (Schiefelbein et al. 2013; Ukkonen et al. 2013; Gomez et al. 2017). Therefore, this study is aimed at providing an overview of results with several flask designs and microwell plate configurations for the cultivation of *R. eutropha* with WAF to produce PHAs. It further reports investigations with respect to a changing fat solubility due to lipase secretion during microbial growth. Due to consumption, the lipid concentration decreases, which leads to dynamically changing conditions. Steady states with model fluids and without microorganisms, in contrast, might not lead to comparable conditions. The achievable reproducibility concerning growth and product formation is determined and compared to fat free cultivation conditions which are present if, e.g., water-soluble carbohydrates are applied. Finally, a workflow is presented that yields optimal cultivation conditions and allows process optimization with WAF supplemented media in shake flasks and microwell plates.

## Material and methods

### Bacterial strain

The wild-type *Ralstonia eutropha* H16 (DSM No. 428, supplied by Leibniz Institute DSMZ-German Collection of Microorganisms and Cell Cultures) was used in this study.

### Waste animal fats as carbon feedstock for PHA production

The company ANiMOX GmbH (Germany) provided different WAF products, which as such are by-products of a high pressure thermolysis process of protein separation from animal waste streams. In this study, WAF from fish, chicken, and pork were investigated. The distribution of fatty acids within the WAF, their physical properties, and content of each “ANiFAT” product are shown in Table 1.

**Table 1:** Physical properties, content of waste animal fats, and distribution of fatty acids in percentage (wt). *FFA*, free fatty acids; *WAF*, waste animal fat. ^a^Room temperature

WAF	Melting point (°C)	Content (wt%)				Fatty acids^1^			
		Protein	Fat	FFA	C14:0	C16:0	C16:1	C18:0	C18:1-3
ANiFAT_F	RT ^a^	1.31	68.1	5.53		27.28	2.11	2.35	45.72
ANiFAT_C	34	0.19	99.8	3.68	0.75	27.52	4.20	6.02	57.86
ANiFAT_P	45	0.13	99.8	1.93	1.20	25.42	2.67	9.89	60.81
ANiFAT_P FFA	60	3.38	95.6	51.8		21.39	1.92	33.89	42.55

^1^Docosahexaenoic acid and eicosapentaenoic acid, which might be detectable in fish, were not included in the table.

### Growth media and cultivation conditions in shake flasks

As media, dextrose-free tryptic soy broth (TSB; Carl Roth GmbH & Co. KG, Germany) was used for overnight cultivations. All growth media contained 10 μg mL^-1^ gentamicin sulfate to prevent contamination. Phosphate-buffered minimal medium, as described previously, was used for all experiments, with an initial pH value of 6.8 (Santolin et al. 2021). In minimal medium cultivations, nitrogen limitation was applied to trigger maximum PHA accumulation. Urea (Carl Roth) was used as nitrogen source. WAF, as shown in Table 1, were used as a carbon source. *R. eutropha* was initially grown overnight in 10 mL TSB from a single colony in a 125 mL UltraYield^TM^ flask (U.Y.F.), which was covered with a sterile AirOtop^®^ membrane (both obtained from Thomson Instrument Company, CA) at 200 rpm and 30 °C. Cells were centrifuged at 5,000 rpm for 5 min at 4 °C, and the pellet was resuspended in 0.85 wt% saline prior to inoculation to an initial OD_600_ of ~0.05 in 50 mL or 100 mL flask cultures, containing 1–3 wt% WAF and 0.056 wt% urea, respectively.

### Growth and PHA production in dependence on the shake flask design

Growth and PHA production were compared in various shake flask designs, as shown in Table 2: (i) Sensor Flask SFS-HP5-PSt3—single-use polycarbonate flasks (PSC bottom-baffled, PSC w/o baffles - PreSens Precision Sensing GmbH, Germany); (ii) U.Y.F.—single-use polypropylene flask (see Section 2.3, UYF 500 mL, UYF 250 mL, Thomson Instrument Company); (iii) TubeSpin Bioreactor 600—single-use polypropylene flask (TS - TPP^®^ Techno Plastic Products AG, Switzerland); (iv) DURAN^®^ bottom-baffled flask with GL 45 thread—re-usable glass flask (DN - DWK Life Sciences GmbH, Germany); and (v) DURAN side-baffled Erlenmeyer flask—re-usable glass flask, wide neck with two GL 25 and one GL 18 ports (DN side-baffled - DWK), respectively. The UYF, TS, DN, and DN side-baffled flasks were closed on top using 0.2 μm re-sealable and sterile AirOtop membranes, to provide a high gas exchange during cultivations. The flasks were autoclaved with aluminum foil. The AirOtop membrane was placed on flasks prior inoculation. The DN was alternatively closed with a 0.2 μm re-usable PTFE membrane screw cap, which was autoclaved separately (“PTFE-A”) or with the flask and media (“PTFE-B”). For all shake flasks beside the sensor flasks equipped with the PreSens system, the dissolved oxygen (DO) concentrations were measured in-line with a polarographic electrode (Medorex e.K., Germany) through a self-made and sealed hole in the corresponding flask walls. Data was recorded on-line with the wireless sensor system SENBIT (TeleBITcom GmbH, Germany). The filling volume was kept at 20% of the maximum volume, except for TS (16 %) and SENBIT Erlenmeyer flasks (10%), due to practical reasons. A shaking amplitude of 50 mm was applied.

**Table 2:**
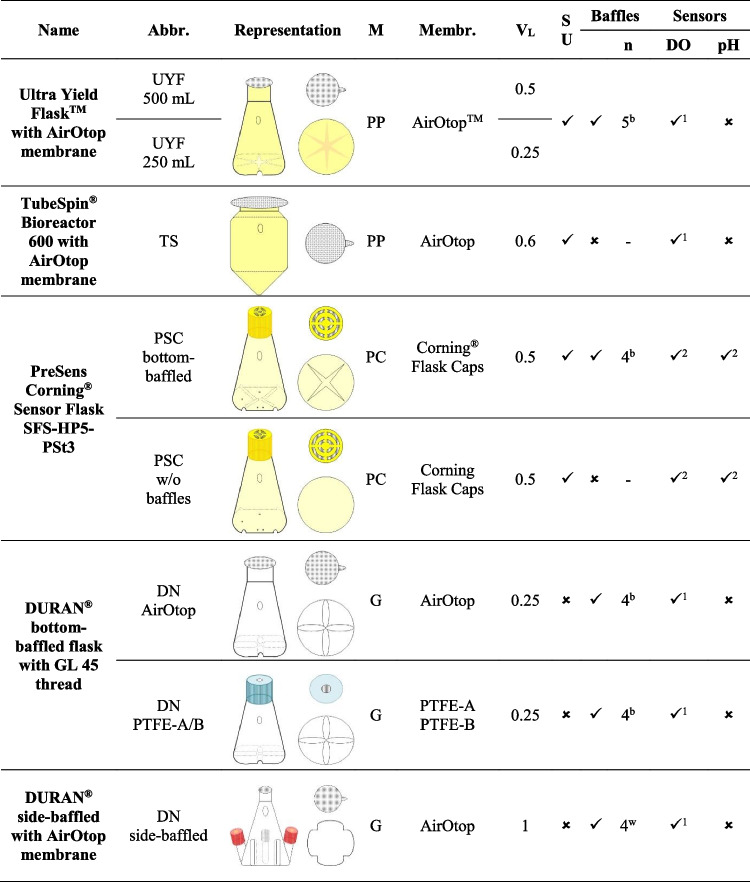
Description of shake flasks. *M*, material; *G*, glass; *PP*, polypropylene; *PC*, polycarbonate; *Membr.*, membrane; *V*_*L*_, volume [L]; *SU*, single-use; *n*, number. ^1^Measured in-line with polarographic electrode, ^2^measured in-line via sensor spots at the bottom of the flask, ^b^baffles at the bottom, and ^w^baffles on the walls

Shake flasks were inoculated from TSB overnight cultures to an initial OD_600_ of approx. 0.05. The minimal medium contained 0.056 wt% urea as a nitrogen source and 1 wt% non-emulsified WAF (ANiFAT_P). The shake flasks were incubated at 200 rpm and 30 °C for 72 h. Shake flask experiments were performed in duplicate cultivations.

### WAF emulsifications

The influence of the emulsifiers was studied by growing *R. eutropha* in 10 mL TSB with a concentration of 1.5 wt% of each emulsifier for 24 h. Cultivation conditions were the same as for the TSB pre-cultures. For WAF emulsification, two different methods, namely, non-mechanical and mechanical treatment, were tested. The homogenization with mechanical treatment was performed with pre-heated solutions at 50 °C as follows: sodium phosphate, potassium sulfate, distilled water emulsifier, and WAF were combined and emulsified by homogenizing with an Ultra-Turrax T25 (IKA^®^-Werke GmbH & Co. KG, Germany) for 1 min at 8,000 rpm. After emulsifying the WAF, the medium was autoclaved for 20 min at 121 °C. After cooling down to room temperature, the remaining media compounds were added from sterile stocks. In case of the non-mechanical method, WAF, emulsifier, phosphate buffer, potassium sulfate, and distilled water were autoclaved. All other media components were added separately (see above).

### Microwell plate cultivation of *R. eutropha* with WAF

24-microwell plates with typical circular wells and a maximum volume of 3 mL (OxoDish^®^ OD24, PreSens) and 24-deepwell plates with rectangular wells and a maximum volume of 11 mL (OxoDish OD24-DW, PreSens) were used in this study for the plate cultivation scale as described in the result section. A shaking amplitude of 50 mm for well plate cultivations was applied. So-called “sandwich covers” (Duetz et al. 2000) as recommended for aerobic bacterial cultivations for each respective plates (Enzyscreen BV, The Netherlands) were used.

A DoE approach was performed to optimize the cultivation conditions in the 24-microwell plates with circular wells. Each microwell had an oxygen sensor spot at the bottom. Three factors were varied: (i) the concentration of the emulsifier, (ii) the filling volume, and (iii) the shaking speed with two levels each (3^2^ factorial design, Table 3). The center point was performed in triplicate. To ensure identical cultivation conditions for the microwell replicates, 50 mL of each media was inoculated from TSB overnight cultures (see above). Then, either 1 or 2 mL was transferred to each of the microwells. For media preparation, between 1.5 and 3 wt% ANiFAT_F, ANiFAT_C, ANiFAT_P, and ANiFAT_P free fatty acids were pre-emulsified either with gum arabic (Carl Roth), emulsan (Dragonspice Naturwaren, Germany), or mannoprotein (La Littorale, France) in concentrations of between 0.1 and 3.5 wt%, using either mechanical or non-mechanical pre-emulsification. The program MODDE Pro 11 (MKS Umetrics AB, Sweden) was used for data analysis. The experiment was repeated with wider parameter ranges (Table 4) based on the results shown in Fig. 6 A1–3.

**Table 3:** Design of experiments with three factors on two levels (3^2^)

RPM	Volume (mL)	Concentration of emulsifier agents (wt%)	
		Gum arabic, mannoprotein	Emulsan
250	1.0	1.5	0.5
300	1.0	1.5	0.5
250	2.0	1.5	0.5
300	2.0	1.5	0.5
250	1.0	2.5	3.0
300	1.0	2.5	3.0
250	2.0	2.5	3.0
300	2.0	2.5	3.0
250/300	1.5	2.0	1.5
250/300	1.5	2.0	1.5
250/300	1.5	2.0	1.5

**Table 4:** Parameters for boundary expansion of results from experimental design

Emulsifier	RPM	Concentration of emulsifier (wt%)
Gum arabic	200	2.0
		2.5
		3.0
	250	
	300	
Emulsan	300	0.1
		0.25
Mannoprotein	300	3.0
		3.5

### Analytical methods

For cell dry weight (CDW) determination, 1 mL of culture broth was transferred into a 1.5 mL pre-weighted conical Eppendorf tube, which was centrifuged at 4 °C and 5,000 rpm for 5 min. The supernatant was discarded, and pellets were washed twice; first, with 1 mL of cold water (4 °C); and second, with a mixture of cold water (70 wt%) and cold hexane (30 wt%) in order to remove residual lipids. The washed pellet was centrifuged again and dried in an oven at 75 °C until dryness. High-performance liquid chromatography with diode array detection (HPLC-DAD 1200 series, Agilent technologies, Germany) was used to determine the polyhydroxybutyrate (PHB) content from dried *R. eutropha* H16 cells using the crotonate assay protocol, which was adapted from Karr et al. (Karr et al. 1983), as described previously (Gutschmann et al. 2019). Physical properties, fat content, and fatty acid distribution of the WAF where determined as described previously (Riedel et al. 2015).

## Results

The aim of this study was to evaluate different shake flask and microwell plate designs and cultivation conditions to obtain a suitable workflow under typically dynamic cultivation conditions when using WAF as media component in *R. eutropha* cultivations. WAF were used as carbon feedstock due to their low price and high availability on the market. Since the melting point of WAF was higher than the cultivation temperature, it was challenging to apply these hydrophobic carbon sources in an aqueous medium on a small scale. Therefore, different emulsification strategies were evaluated to enhance the bioavailability of the WAF in microwell plates. Finally, a workflow for the cultivation in shake flasks and microwell plates is proposed.

### Evaluation of shake flask geometries for growth and PHA production

Six different shake flask designs, equipped with different caps and membranes for the oxygen transfer, were applied to compare growth and PHB production of *R. eutropha* H16. An emphasis was put on commercially available systems to ensure practical relevance. Each design is described in Table 2. Minimal media with 1 wt% ANiFAT_C and 0.056 wt% urea were used for all cultivations. Either tailor-made or commercialized analytical tools for in-line determination of the dissolved oxygen (DO) concentration as one of the most decisive parameters for a suitable cultivation system were used to monitor he time course throughout the cultivation. This made it possible to evaluate whether the gas mass transfer is sufficient for *R. eutropha* cultivations.

Shake flasks vary typically in design and cap structures, which both influence process performance, especially if the oxygen supply is a critical factor. The gas exchange between the inner head space and the surrounding environment for maintaining a certain oxygen concentration and avoiding a carbon dioxide accumulation depends on the membrane material and thickness. It further depends on the exchange area and its dampness, e.g., caused by splashing water droplets. In a previous study, bottom-baffled plastic flasks with thin cellulolytic membranes (here UYF) showed a considerably higher volumetric gas mass transfer as other systems (Glazyrina et al. 2011). Therefore, first, several flasks with baffles at the bottom, but different caps were investigated; second, flasks with baffles at the side; and finally, flasks without any baffles. The latter create less turbulence, but usually a larger liquid layer on the side walls in order to achieve a compatible gas mass transfer.

**Fig. 1:**
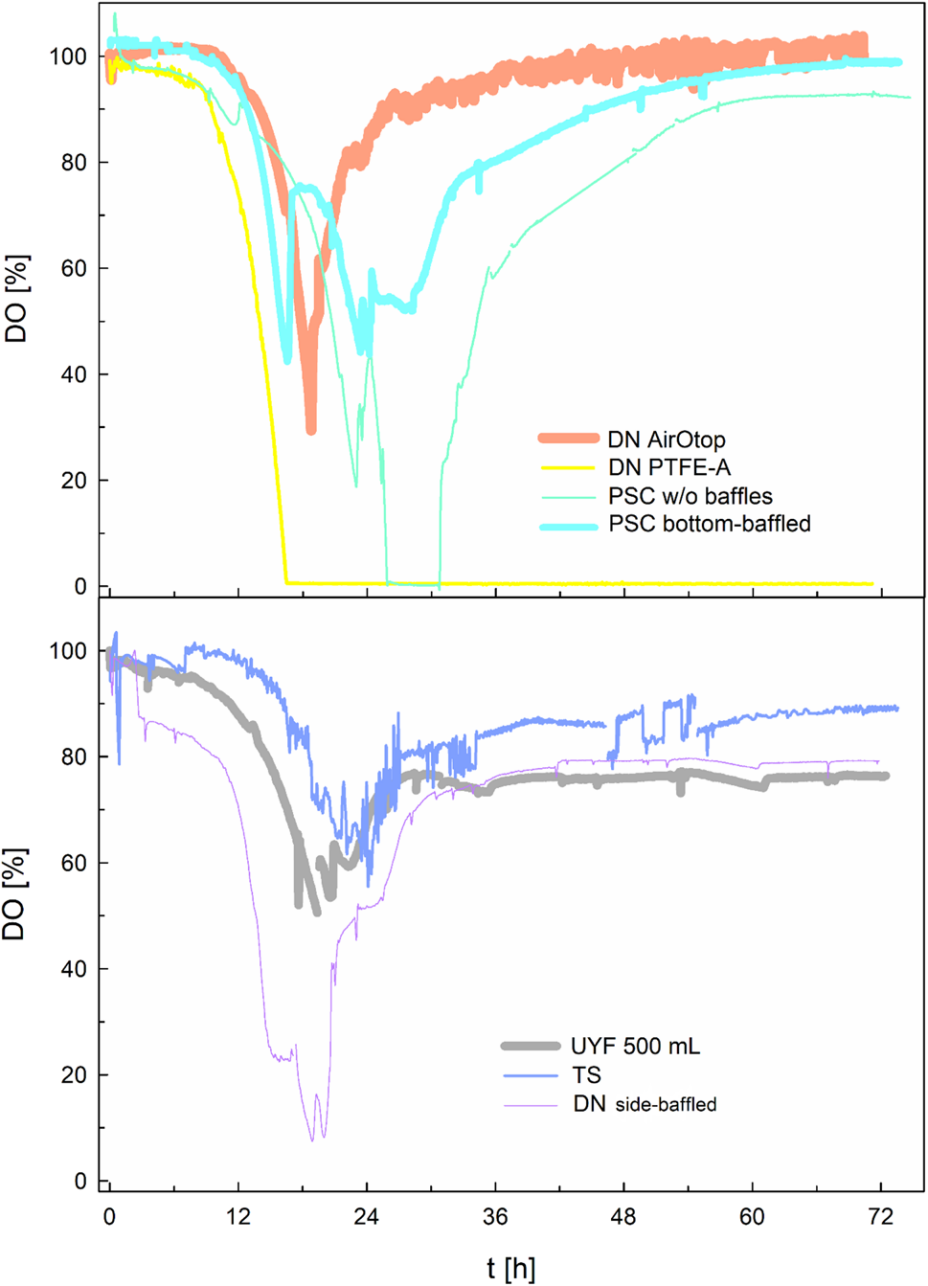
In-line dissolved oxygen concentrations over the course of shake flask cultivations. *Ralstonia eutropha* H16 was grown in minimal medium with 1 wt% ANiFAT_C as carbon and 0.056 wt% urea as nitrogen source. Cultivations were conducted for 72 h at 30 °C and 200 rpm. DO, dissolved oxygen concentration. Descriptions of the abbreviated shake flask design variants are shown in Table 2

**Fig. 2:**
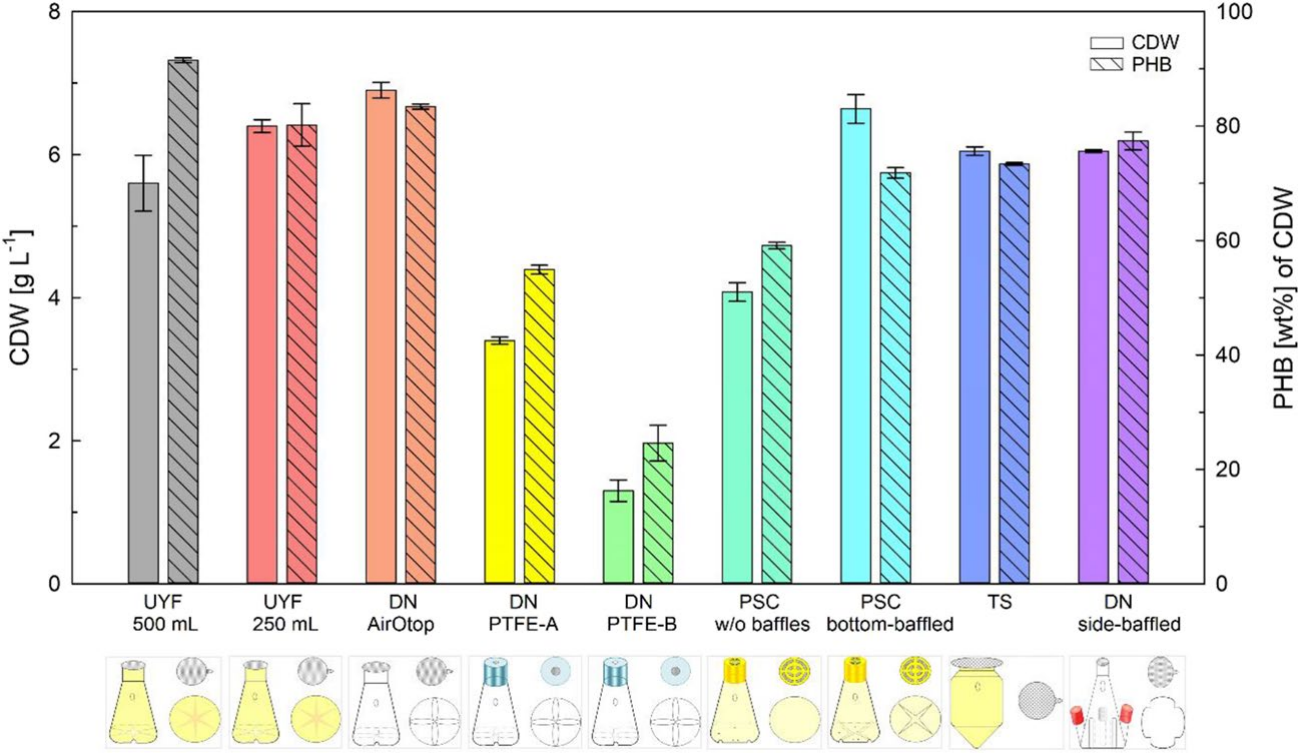
Final cell dry weight concentration and polyhydroxybutyrate accumulation of *Ralstonia eutropha* H16 shake flask cultivations. *R. eutropha* H16 was grown in minimal medium with 1 wt% ANiFAT_C as carbon and 0.056 wt% urea as nitrogen source. The different types of shake flasks were cultivated for 72 h at 30 °C and 200 rpm. Mean values from duplicate experiments are shown. Error bars show the span between minimum and maximum values

As expected, the shake flask and cap designs had a strong impact on the DO concentration in *R. eutropha* batch cultivations (Fig. 1). One of the unbaffled flask design (PSC w/o baffles) was not able to provide a sufficient k_L_a-value to maintain the culture at DO excess. Cultures in flasks with a narrow PTFE membrane area (DN PTFE) were opposed to a strong oxygen limitation from a certain timepoint on, although these flasks were equipped with baffles on the bottom. All other bottom-baffled flasks competed better than the side-baffled flask (DN side-baffled). There, the DO remained above 20% of saturation, which is widely seen as a value at which no oxygen limitation occurs inside a cell. The impact of DO limitation was clearly seen at the achievable biomass yield: all cultivations without oxygen limitation reached a final CDW concentration between 5.6 and 6.9 g L^-1^ with a PHB content of 72 and 92 wt% per CDW. Among them, cultivations in bottom-baffled flasks with wide necks and concomitantly high exchange areas reached the highest biomass concentration (UYF, DN AirOtop). Cultivations exposed to oxygen limitation reached only a CDW of 1.3 to 4.1 g L^-1^ and showed substantially lower PHB accumulation of only 24.6 to 59.5 wt% per CDW (e.g., DN PTFE). Highest specific PHB concentrations, in contrast, were achieved in UYF 500 mL (Fig. 2). One of the reasons for the worse process performance of the DN PTFE flasks were wet membranes from autoclavation of flasks with media in it. If caps were autoclaved separately and dried (“PTFE-A”), a 2.5-fold increase in biomass and a 2.2-fold increase in PHB accumulation were achieved (Fig. 2). Nevertheless, the yields remained lower than with other flask designs. Interestingly, the TS design (that is a design with a cylindroconical bottom and no baffles) yielded very good results similar to the UYF. Gas mass transfer is usually comparably high if a water spout forms (Maier et al. 2004). It creates a larger surface area of the liquid phase and, more importantly, a higher level of turbulence at the edges between the conical bottom and the vertical vessel walls. As a large membrane area was applied in our study, it can be assumed that the membrane area had an equal or even larger effect on a sufficient dissolved oxygen supply than the presence of baffles. Even in case the DO dropped below 20 % of saturation, but only shortly below 10% (as for DN side-baffled), CDW and PHB formation was hardly affected. Results were similar, so that several shake flask designs in combination with the membranes applied in our study seem to be suitable. Also, no tremendous differences were seen in the reproducibility of results. It is noteworthy to mention, however, that they were highest in TS.

The type of membrane had a big effect on the evaporation of the culture media. UYF and DN AirOtop lost 28% of water (in average) over the course of the experiment. The cultivations in the TS even lost 60% of water, whereas the loss of the DN side-baffled was only 10%. Evaporation was significantly lower in cultivations with PTFE membranes (3% at smallest membrane diameter, DN PTFE). In PSC, 2% was lost in baffled and 14% in the unbaffled design. In all experiments, the final culture volumes and, thus, the final CDW values were corrected for evaporation losses.

In order to examine film formation, the portion of WAF, which stuck to the wall of each flask, was quantified by mass determination after the liquid suspension had been removed, and the remaining fat was entirely collected. While about 0.1 g of WAF was collected from TS (less than 1 % at duplicate experiments), the amount of WAF collected from the wall of other types of shake flasks was much higher. Highest amounts were collected from UYF and PSC: up to 0.28 g of fat was determined. In this case, the variation between duplicates exceeded 100%. This indicates an issue when such designs are used, as the amount of fat that sticks to the wall is neither negligible nor reproducible. It would require numerous repetitions to achieve statistical validity of experimental results.

### Evaluation of emulsifier agents

**Fig. 3:**
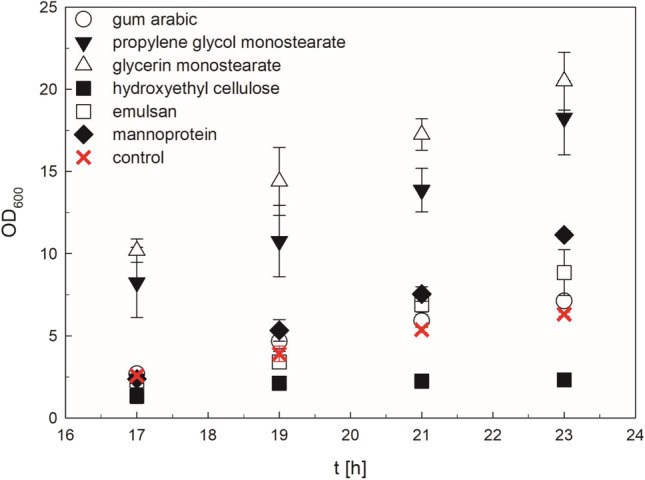
Determination of the influence of emulsifying agents on the growth of wild-type *Ralstonia eutropha* H16. Bacteria were grown for 24 h in TSB medium, supplemented with 1.5 wt% of each emulsifying agent in shaking flasks. Error bars are indicating the standard deviation of the arithmetic mean from triplicate cultures. (Data are explicitly shown in the supplementary Table S1)

Formation of a fat film can particularly happen under flow conditions of low turbulence, as they are often experienced in microwell plates. In order to prevent fat material sticking on the walls, the use of emulsifying agents was investigated. The influence of emulsifying agents on the growth of *R. eutropha* and on the reproducibility of cultivations was examined. TSB cultures, inoculated from single colonies as described in the methods section, were supplemented with 1.5 wt% of each of the following emulsifiers: propylene glycol monostearate, glycerin monostearate, hydroxyethyl cellulose, mannoprotein, emulsan, and gum arabic. The OD_600_ was measured during the exponential growth phase of *R. eutropha* (Fig. 3, Supplemental Table 1). Propylene glycol monostearate and glycerin monostearate cultures showed significantly higher optical densities than the control. Both serve also as a carbon source for *R. eutropha* and, thus, influence the experimental outcome. Cultures supplemented with hydroxyethyl cellulose grew slower than the control. The growth with gum arabic and emulsan cultures was nearly indistinguishable from the TSB control culture. Cultures with mannoprotein achieved also similar growth as a control throughout 19 h. Based on these results, the emulsifiers, gum arabic, emulsan, and mannoprotein were chosen for subsequent experiments.

**Fig. 4:**
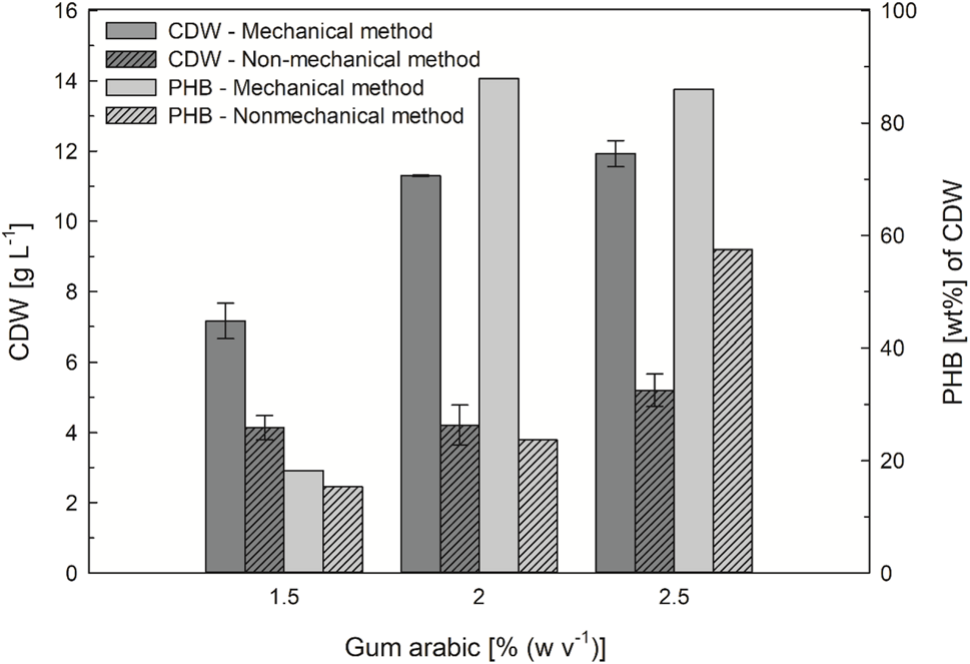
Comparison of the mechanical and non-mechanical pre-emulsification methods for waste animal fats. 3 wt% ANiFAT_P was pre-emulsified with 1.5–2.5 wt% gum arabic. *Ralstonia eutropha* H16 was grown for 72 h at 30 °C and 300 rpm in microwell plates. All cultivations were performed in triplicates. Error bars are indicating the standard deviation. The content of polyhydroxybutyrate (PHB) per cell dry weight was measured after pooling the biomass from the triplicate cultivations

WAFs were pre-emulsified with each of the different emulsifiers to enhance their bioavailability, according to Riedel et al. (Riedel et al. 2015). The application of the mechanical emulsification method led to significantly higher cell growth and PHB accumulation, in comparison to the non-mechanical emulsification method, as seen in Fig. 4 (11.93 g L^-1^ CDW and 86.02 wt% PHB per CDW) vs. 5.2 g L^-1^ CDW and 57.49 wt% PHB per CDW, respectively). Therefore, the mechanical emulsification method was chosen to pre-emulsify the WAF for further experiments.

### Identification of suitable microwell plate cultivation conditions

In order to compare process performance between the shake flask and well plate scale and to identify a suitable design for conducting cultivations with WAF in microwell plates, two different designs of cavities were studied: (i) rectangular deep-wells and (ii) round-shaped wells, both on a 24 microwell plate format. When applying between 0.5 and 4.5 wt% ANiFAT_C and working volumes of between 1 and 3 mL, a high variability of values was observed in first experiments (data not shown). The variation between replicates was significantly higher than 5%. Evaporation depended on the WAF content: experiments with 3 wt% WAF showed the lowest evaporation (5 ± 3%) independent from the filling volume. Since WAF material heavily stuck to the walls of the corners of the rectangular shaped wells, round shaped microwell plates with a working volume of 1 and 2 mL were applied in subsequent experiments.

**Fig. 5:**
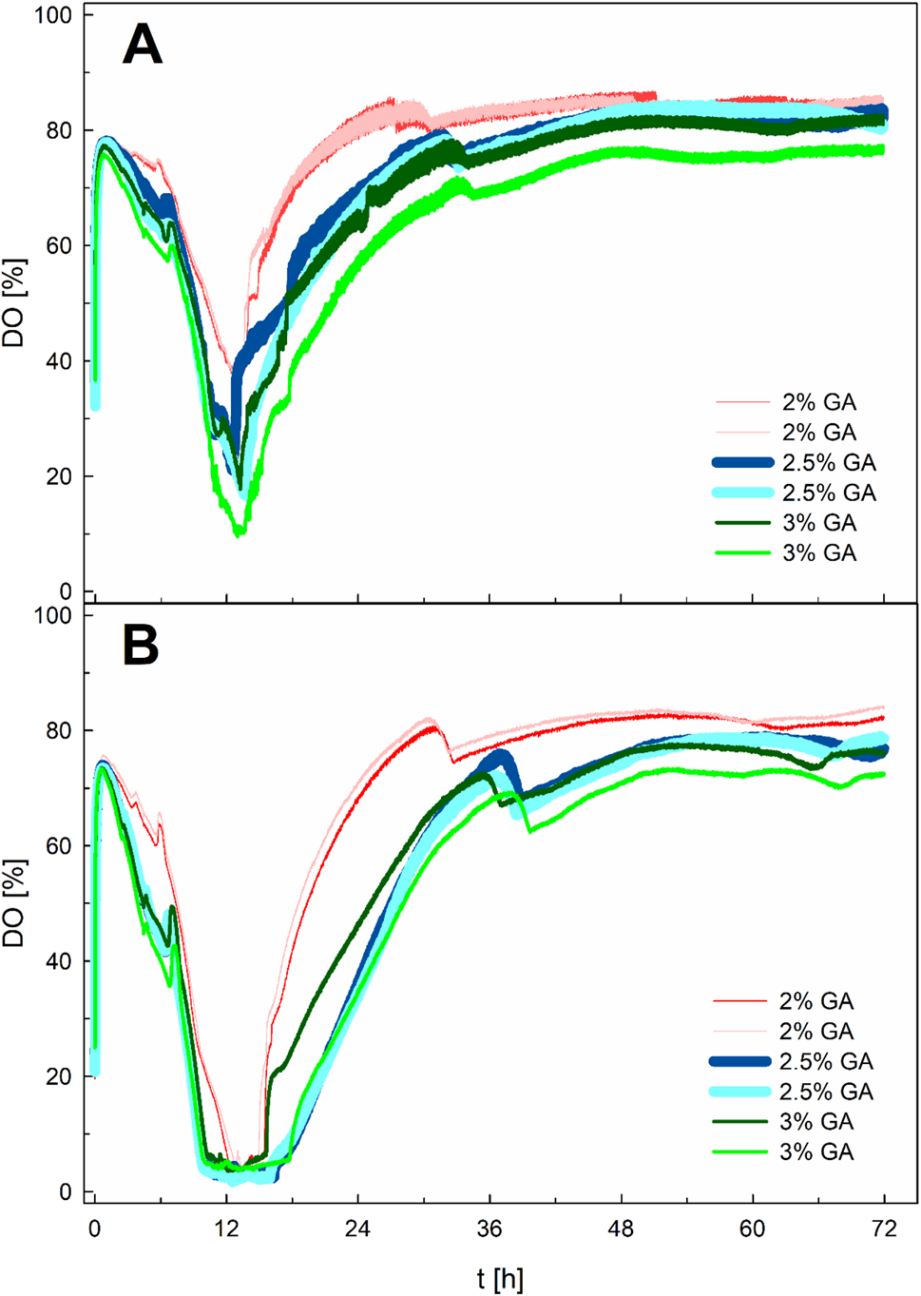
Dissolved oxygen (DO) concentration during *Ralstonia eutropha* H16 microwell plate cultivations. Filling volume of 1 mL (A) and 2 mL (B). Between 2 and 3 wt% gum arabic (GA) was used for the pre-emulsification of the 3 wt% ANiFAT_P. Microwell plates were cultivated for 72 h at 30 °C and 250 rpm

Gum arabic, mannoprotein, and emulsan were tested for cultures with ANiFAT_P according to the experimental design (design of experiments - DoE) set-up described in the method section and shown in Table 3. Growth (CDW), specific PHB accumulation, and DO values were measured during these microwell plate cultivations. As shown in Fig. 5, all cultivations with a working volume of 2 mL exhibited DO limitation. Therefore, only results from cultivations with a working volume of 1 mL were used to optimize a workflow protocol for plate scale cultivations.

**Fig. 6:**
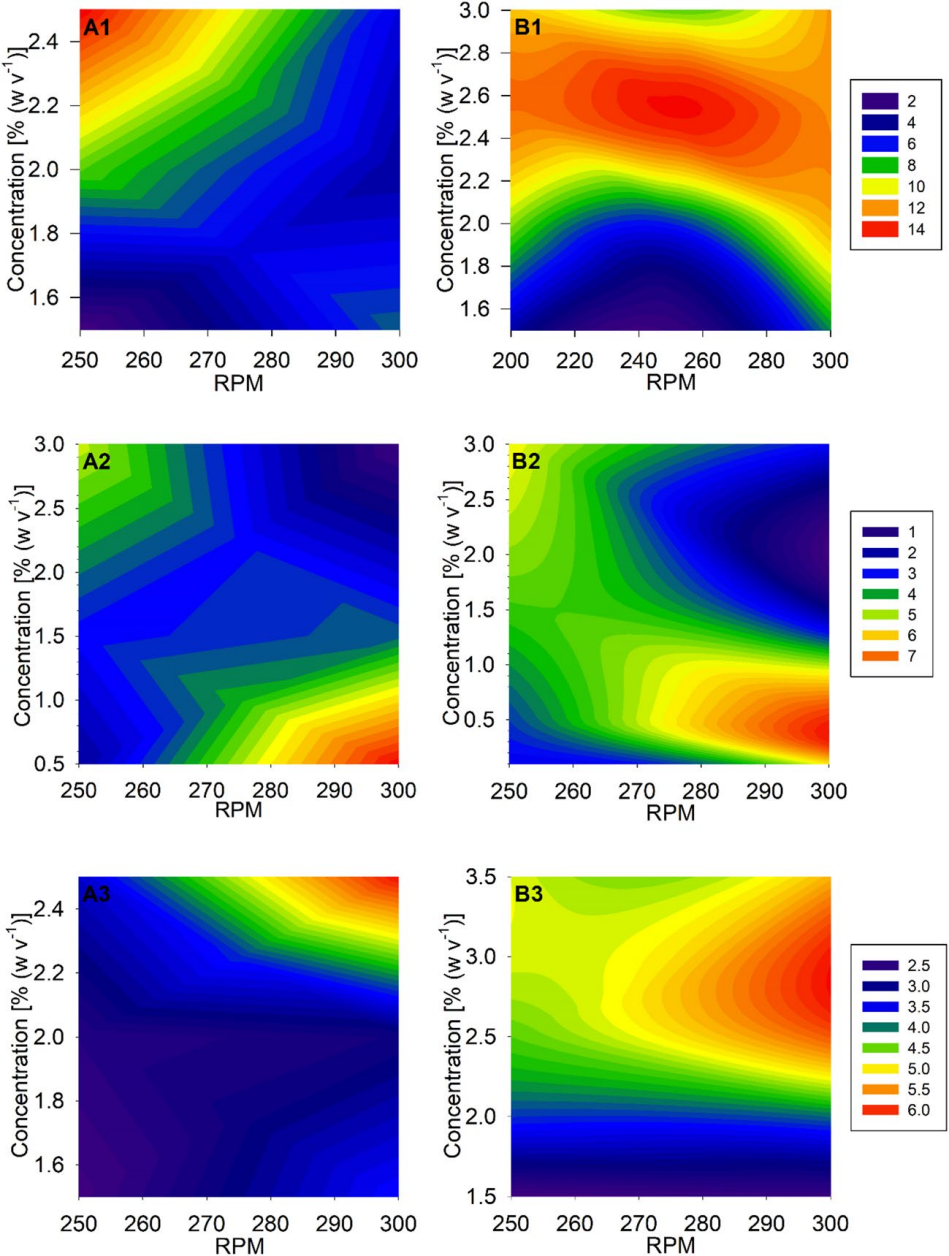
Optimization of the microwell plate culture conditions for *Ralstonia eutropha* H16 grown on waste animal fats. Gum arabic (A1, B1), emulsan (A2, B2), and mannoprotein (A3, B3) were used as emulsifying agents in various concentrations. *Ralstonia eutropha* H16 was grown for 72 h in minimal medium with 0.056 wt% urea as a nitrogen source and 3 wt% mechanical pre-emulsified ANiFAT_P as carbon source at 30 °C at various shaking speeds. Part A shows the results according to the DoE set up. Part B shows the repeated results with wider parameter borders. The cell dry weight concentrations (g L^-1^) at different growth conditions are visualized through the given color code

The type and concentration of the applied emulsifier and shaking speed of the microwell plate had a substantial influence on cell growth and PHB production (Fig. 6). Best results for each emulsifier were obtained at boundary conditions of the DoE (Fig. 6a). In order to investigate the border regions of the DoE, another set of experiments with wider boundary conditions was performed as described in the methods section. These experiments (Fig. 6b) confirmed the results of the first set-up. Overall, best results were achieved with 2.5 wt% gum arabic, a working volume of 1 mL, and a shaking speed of 250 rpm, resulting in 14 g L^-1^ CDW with 70 wt% PHB per CDW. The best results were achieved with 0.5 wt% emulsan and a shaking speed of 300 rpm, resulting in 7.53 g L^-1^ CDW with 59 wt% PHB per CDW. 2.5 wt% mannoprotein and a shaking speed of 300 rpm resulted in 7 g L^-1^ CDW with 20 wt% PHB per CDW.

### Cell growth and PHB accumulation from different WAF

**Fig. 7:**
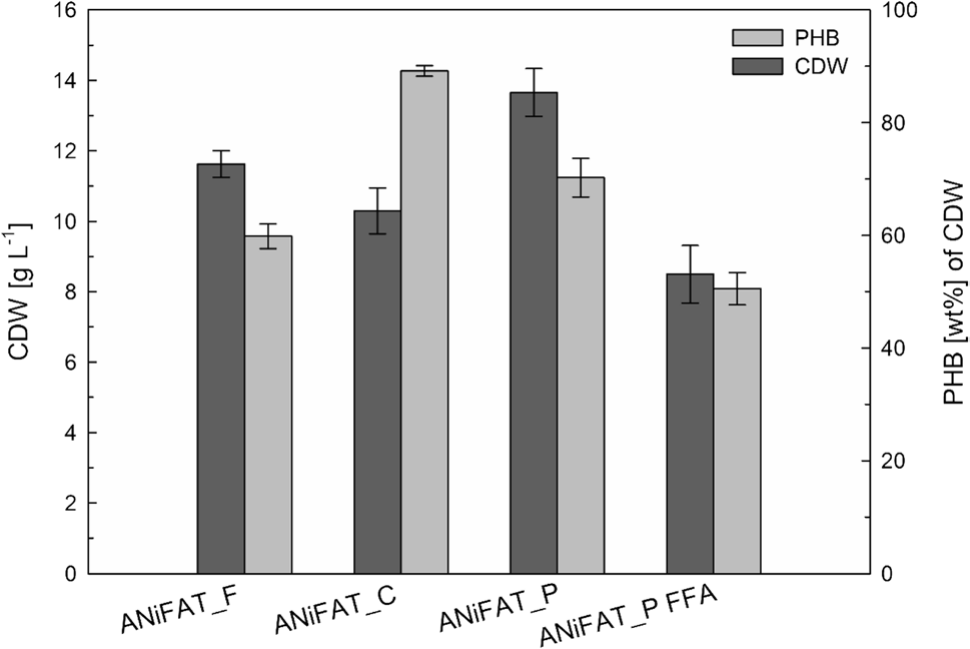
Comparison of cell growth and PHB accumulation in microwell plates from different waste animal fats. *Ralstonia eutropha* H16 cultures were performed for 72 h at 30 °C, and 1 mL minimal medium with 0.056 wt% urea as nitrogen source and 3 wt% mechanically pre-emulsified waste animal fats with 2.5 wt% gum arabic was used as carbon source. All cultivations were performed in triplicates. Error bars indicate the standard deviation

PHB production with ANiFAT_F, ANiFAT_C, ANiFAT_P, and ANiFAT_P FFA was compared in microwell plates under optimal cultivation conditions as previously described in order to investigate the influence of the fat type (and different melting temperature) on the reproducibility (Fig. 7). The experiment showed significant improvement of growth and PHB production using our established pre-emulsification conditions. The highest biomass was obtained with ANiFAT_P (13.6 ± 0.7 g L^-1^), whereas the highest PHB accumulation (89.2 ± 0.9 wt%) was reached with ANiFAT_C as carbon source. Cultivations with WAF with the highest free fatty acid content, ANiFAT _P FFA, resulted in the lowest PHB accumulation (50.5 ± 2.8 wt%) and a final CDW of only 8.5 ± 0.8 g L^-1^. In all cases, the reproducibility was sufficient to distinguish between the process outcomes.

## Discussion

In this study, the various shake flask designs showed distinguished features in terms of reproducibility for WAF-based PHB production with *R. eutropha*. Similarly, tremendous differences were observed for microwell plate designs. Best results in shake flasks were obtained with flasks, which had a comparably high membrane area for gas exchange with the surrounding environment (Fig. 2). The increase of the gas exchange seems to be more important than the presence or location of baffles as the TS design competed well with bottom-baffled flask designs. Results confirmed previous investigations that UYF provided a comparably high k_L_a-value of over 200 h^-1^, which was at least twice as high as k_L_a-values determined in Erlenmeyer glass flasks in the same study (Glazyrina et al. 2011) and also for TS elsewhere (Monteil et al. 2013; Zhu et al. 2017). k_L_a-values of UYF are in a similar range than values obtained from experiments in bottom-baffled Erlenmeyer flasks with PTFE membranes (Schiefelbein et al. 2013). This is in agreement to our study, in which growth and product formation are similar in UYF and PSC with baffles. The oxygen mass transfer might be comparably higher due to a larger surface area of the hydrophobic phase with better oxygen solubility in flasks, in which film formation contributes substantially to the k_L_a-value. It was shown that film formation, which is related to the viscosity, contributes to the k_L_a-value in unbaffled flasks (Maier et al. 2004; Lattermann and Büchs 2015). In case baffles contribute to a small droplet size by lipid droplet destruction, a similar effect of a large hydrophobic surface is created, which can have a positive effect on the k_L_a-value, too. Such effects had been observed before with other hydrophobic additives in shake flask cultures (Pilarek et al. 2013). What design parameter is dominant to support oxygen gas mass transfer, however, can change throughout the cultivation when the organism secretes lipases, the hydrophobic fraction gets smaller and the viscosity increases due to cell growth. Based on studies in fully aerated bioreactors (Gutschmann et al. 2023b), a k_L_a-value of between 30 and 50 h^-1^ might be sufficient at the growth rates and CDW concentrations that occurred in our experiments. In case the oxygen concentration in the headspace is lower than in air, which is usually the case in shake flasks, the k_L_a value that is required to maintain sufficient oxygen supply has to be, however, several fold higher, probably in between 100 and 200 h^-1^.

A sensitive factor for oxygen gas mass transfer is naturally the closure of cultivation flasks (Nikakhtari and Hill 2006). The features of gas mass transfer can change, when lipid containing liquid sticks to the inner site of membranes, then clogging pores for gas transfer. The larger the diameter for gas transfer, the lower is the risk that the membrane is blocked to completion; if not, other design specifications hinder membrane wetting. Hence, the optimal choice of a shake flask for WAF containing media may differ from some assumptions made for purely aqueous cultures. The membrane area was the most important factor for a good process performance in shake flasks in our study.

The reproducibility of the shake flask experiments is also affected by the formation of fat film on the walls, since this fat is not bioavailable for biochemical conversion during the cultivation. Using pre-emulsified WAF can increase the reproducibility in shake flask experiments by reducing fat film formation.

Cultivations in 24-well plates, beside shake flasks, were subject to several investigations to optimize cultivation conditions of *R. eutropha* with WAF-based media. Although the plate scale offers a potential for a faster screening, e.g., through intensified parallelization, the achievement of a reproducible performance of cultivations with WAF in a plate scale is challenging. Due to the high melting temperatures of the WAF (up to 60 °C), *R. eutropha* was not able to emulsify WAF itself in microwell plate cultivations sufficiently. Hence, WAF stack on the walls with subsequently low accessibility for cells. Film formation at the well walls is a common issue and frequently observed, e.g., also for oleaginous organisms (Kosa et al. 2018). The presence of lipophilic components in the media may lead to an intensified film formation, as the hydrophobic nature of plastic surfaces provokes the attachment of fat. Therefore, in our study, emulsifying agents were applied to improve the conversion of WAF to biomass and PHB. It was shown previously that a pre-emulsification strategy is a useful method for increasing the surface area of plant oils for a more effective growth (Budde et al. 2011). We therefore evaluated the influence on growth of *R. eutropha* with several emulsifying agents. Propylene glycol monostearate and glycerin monostearate did not inhibit cell growth, but they served as an additional carbon source for *R. eutropha*. Both belong to the fatty acid ester group. It is considered that an esterase of *R. eutropha* is able to cleave off the fatty acids from these emulsifying agents. Hydroxyethyl cellulose cultures inhibited the growth of *R. eutropha*. Most probably, the chosen emulsifier concentrations were too high, since they increased visibly the viscosity of the media. Mannoprotein, instead, cannot serve as a carbon source for *R. eutropha*, since the microorganism is unable to utilize mannose, the sole carbon source in mannoprotein (Cameron et al. 1988; Sichwart et al. 2011). Gum arabic (Budde et al. 2011) and emulsan cannot serve as an effective carbon source for *R. eutropha* as well. As expected, no significant difference in the growth profile was seen with these emulsifying agents.

In order to enhance the cultivation reproducibility, an emulsification strategy with mechanical treatment was investigated. The mechanical homogenization method resulted in a suitable oil-in-water emulsion: the high shear rates caused a rapid and intensive dispersion of the inner (oil) phase as well as a highly effective splitting of the oily components into small particles. Their larger interfacial area can be covered with the emulsifier gum arabic. The mechanically created emulsion was stable during the whole cultivation time and prevented fat film formation in the wells. Although suitable plate closure devices were applied to prevent wetting of them (Duetz et al. 2000), the risk of clogged pores of membranes was further reduced by this choice of cultivation mode. The increased bioavailability in the pre-emulsified WAF cultivations resulted in a more than doubled biomass concentration and PHB accumulation, compared to cultivations with non-mechanical pretreatment and with a considerable reproducibility (Fig. 4).

In 2 mL cultures, oxygen limitation occurred in contrast to cultures with a working volume of 1 mL (Fig. 5). k_L_a-values in plates are in a range below 50 h^-1^; if not, rpm values beyond 300 and high shaking diameters are applied, as determined for 96-well plates (Hermann et al. 2003). The k_L_a-values for similar plate designs as used in this study were even lower, by a factor between 5 and 10 (Zhang et al. 2008). In this case, a lower culture volume allowed a better oxygen supply, due to a higher surface to volume ratio when a water spout forms at the well walls. Additionally, the headspace in the 2 mL cultivations was obviously not sufficient for a good gas mass transfer.

To the best of our knowledge, this was the first study, which examines different shake flask designs, as well as microwell plates for the application in process development with WAF as substrate. The nature of this feedstock may require certain constraints. As results show, the membrane area is the most important distinguishing feature among the tested shake-flask designs. Among the unbaffled variants, TS yielded comparable results to bottom-baffled flasks. It would be interesting to investigate whether a combination of the cylindroconical design and the integration of baffles as investigated by Lu et al. would lead to a further improvement (Lu et al. 2021). While several flask designs showed suitability, the application of WAF as substrate in the microwell scale is much more challenging. Only the use of a round-shaped well format and mechanical pre-emulsification with gum arabic led to repeatable cultivation results. Nevertheless, the final workflow proved to be reliable among different WAF. *R. eutropha* cultivations in microwell plates with emulsified WAF and 1 mL culture volume showed a high reproducibility in cell growth and PHB production. Cells accumulated high amounts of PHB up to concentrations of between 70 and 90 wt% per CDW. The maximum cell growth and PHB production were dependent on the type and concentration of the emulsifier and the shaking speed. The method allows the conduction of a large number of parallel experiments, if recent developments in instrumentation and automation in such scales are considered (Takahashi and Aoyagi 2018). The findings can also be valuable for other bioprocesses beyond PHA production when hydrophobic carbon sources are used.

## Supplementary information

ESM 1 (PDF 115 kb)

## Data Availability

Data is available from the authors upon reasonable request.
