# Supplementary material for: Workflow for shake flask and plate cultivations with fats for polyhydroxyalkanoate bioproduction
Source: Appl Microbiol Biotechnol. 2023 Jun 2;107(14):4493–505. doi: 10.1007/s00253-023-12599-w (PMC10313549; doi:10.1007/s00253-023-12599-w)
Supplement: Supplementary file 1 — (PDF 115 kb) [file 253_2023_12599_MOESM1_ESM.pdf]

Supplementary Material for

Workflow for shake flask and plate cultivations with fats for polyhydroxyalkanoate bioproduction

Sebastian L. Riedel[1,2], Ewelina N. Donicz[1], Paula Ferré-Aparicio[1], Lara Santolin[1], Anna-Maria Marbà-Ardébol[1], Peter Neubauer[1], Stefan Junne[1,3]*

[1]Technische Universität Berlin, Institute of Biotechnology, Chair of Bioprocess Engineering, Ackerstraße 76, 13355 Berlin, Germany

[2] Berliner Hochschule für Technik, Department VIII – Mechanical Engineering, Event Technology and Process Engineering, Laboratory of Environmental and Bioprocess Engineering, Seestr. 64, 13347 Berlin, Germany

[3]Aalborg University Esbjerg, Department of Chemistry and Bioscience, Niels Bohrs Vej 8, DK-6700 Esbjerg, Denmark

***Correspondence:** Stefan Junne: stefan.junne@tu-berlin.de, sju@bio.aau.dk

1 Supplementary Tables

Supplementary Table S1. **Data from Figure 3.** Determination of the emulsifying agent influence on the growth of wild-type *Ralstonia eutropha* H16. Bacteria were grown for 24 h in TSB medium, supplemented with 1.5 wt% of each emulsifying agent in shaking flasks. Errors are indicating the standard deviation of the arithmetic mean from triplicate cultures.

**Time [h]**	**OD_{600}**						
	**Control**	**Gum arabic**	**Propylene glycol monostearate**	**Glycerin monostearate**	**Hydroxyethyl cellulose**	**Emulsan**	**Mannoprotein**
**17**	2.566 ± 0.165	2.688 ± 0.050	8.245 ± 2.138	10.185 ± 0.713	1.327 ± 0.442	2.250 ± 0.118	2.371 ± 0.280
**19**	3.901 ± 0.480	4.658 ± 0.620	10.770 ± 2.170	14.400 ± 2.070	2.112 ± 0.411	3.400 ± 0.412	5.326 ± 0.664
**21**	5.371 ± 0.170	5.903 ± 0.395	13.870 ± 1.323	17.250 ± 0.961	2.237 ± 0.093	6.900 ± 0.448	7.541 ± 0.443
**23**	6.316 ± 0.125	7.118 ± 0.05	18.270 ± 2.256	20.500 ± 1.762	2.322 ± 0.051	8.850 ± 1.391	11.146 ± 0.245
